# Study Protocol for the Evaluation of the Health Effects of Superblocks in Barcelona: The “Salut Als Carrers” (Health in the Streets) Project

**DOI:** 10.3390/ijerph17082956

**Published:** 2020-04-24

**Authors:** Laia Palència, Brenda Biaani León-Gómez, Xavier Bartoll, Juli Carrere, Elia Díez, Laia Font-Ribera, Anna Gómez, María José López, Marc Marí-Dell’Olmo, Roshanak Mehdipanah, Marta Olabarría, Glòria Pérez, Anna Puig-Ribera, Marc Rico, David Rojas-Rueda, Hugo Vásquez-Vera, Katherine Pérez

**Affiliations:** 1 Agència de Salut Pública de Barcelona (ASPB), 08023 Barcelona, Spain; ext_bbiaani@aspb.cat (B.B.L.-G.); xbartoll@aspb.cat (X.B.); ext_jcarrere@aspb.cat (J.C.); ediez@aspb.cat (E.D.); lfont@aspb.cat (L.F.-R.); agomez@aspb.cat (A.G.); mjlopez@aspb.cat (M.J.L.); mmari@aspb.cat (M.M.-D.); molabarr@aspb.cat (M.O.); gperez@aspb.cat (G.P.); mrico@aspb.cat (M.R.); hvasquez@aspb.cat (H.V.-V.); cperez@aspb.cat (K.P.); 2 CIBER Epidemiología y Salud Pública (CIBERESP), 28029 Madrid, Spain; 3 Institut d’Investigació Biomèdica (IIB Sant Pau), 08041 Barcelona, Spain; 4 Associació Benestar i Desenvolupament (ABD), 08012 Barcelona, Spain; 5 Health Behavior and Health Education, School of Public Health, University of Michigan, Ann Arbor, MI 48109, USA; rmehdipa@umich.edu; 6 Department of Physical Activity Sciences, Centre for Health and Social Care Research, University of Vic-Central University of Catalonia, 08500 Vic, Spain; annam.puig@uvic.cat; 7 Barcelona Institute for Global Health (ISGLOBAL), 08036 Barcelona, Spain; David.Rojas@colostate.edu; 8 Environmental and Radiological Health Sciences, Colorado State University, Fort Collins, CO 80523, USA

**Keywords:** health and well-being, superblocks, urban planning, quasi-experimental, health impact assessment

## Abstract

Superblocks are currently being introduced in Barcelona to respond to the city’s scarcity of green spaces and high levels of air pollution, traffic injuries, and sedentariness. The aim is to calm the streets by reducing the number of square meters dedicated to private vehicles and to reclaim part of this public space for people. Salut als Carrers (Health in the Streets) is a project to evaluate the potential environmental and health effects of the superblock model with an equity perspective in Barcelona. This study aims to explain the various interventions implemented in different neighborhoods in Barcelona and the methods that will be used to evaluate them in a quasi-experimental and health impact assessment (HIA) approaches. Given the complexity of the intervention evaluated, the project employs mixed methodologies. Quantitative methods include: (a) a pre–post health survey of 1200 people randomly selected from the municipal register asked about self-perceived health and quality of life, social support, mental health, mobility, physical activity, neighborhood characteristics, and housing; (b) pre–post environmental measurements, mainly of nitrogen dioxide (NO_2_), particulate matter of less than 10 µm (PM_10_), and particulate matter of less than 2.5 µm (PM_2.5_) and black carbon; (c) pre–post environmental walkability measures using the Microscale Audit of Pedestrian Streetscapes (MAPS) tool; (d) use of public space and physical activity levels using the System for Observing Play and Recreation in Communities (SOPARC), a validated observation tool; (e) pre–post traffic injury measures with a comparison group; and (f) the comparison and integration of pre–post assessment with previous HIAs and the improvement of future HIAs. Qualitative studies will be performed to analyze residents’ perception of these effects by using: (a) various focus groups according to different participant characteristics who are more or less likely to use the superblocks; and (b) a guerrilla ethnography, which is a method that combines ethnographic observation and semi-structured interviews. This study, which evaluates the impact of an ambitious urban-renewal program on health, will help to assess the effectiveness of public policy in terms of health and health inequalities.

## 1. Introduction

### 1.1. The Superblock Model as a Response to Health-Related City Challenges 

Air pollution is a well-known major risk factor for several diseases and premature death [1]. Other important health determinants in urban areas are traffic injuries, sedentary behavior, and lack of green areas [2,3]. To address these issues, many cities are implementing organizational and infrastructure interventions to encourage residents to walk, cycle, and reduce their reliance on driving [4]. Examples of these interventions are complete streets programs (streets that are designed and operated to enable safe access for citizens of all ages, abilities and modes of transport [5]), which have been implemented in many cities in the United States among other places. For example, the London Healthy Streets Approach [6], which aims to improve air quality, reduce congestion, and help make diverse communities greener, healthier and more attractive places to live, work, play and do business. Additionally the New York Sustainable Streets 2009 program [7], composed of 164 actions committed to sustainable streets in terms of safety, mobility, world-class streets, infrastructure, greening, customer service, and global leadership.

Barcelona is a dense and compact Mediterranean city in Spain that shares many health challenges with other cities, such as high air pollution levels, traffic noise, traffic injuries, lack of green areas, and few relationship spaces [8]. The city includes a central area called “Eixample”, formed by a grid of orthogonal streets (creating city blocks) surrounded by what used to be former villages annexed to Barcelona. To address the aforementioned health issues, in May 2016, the Barcelona city council approved the measure “Omplim de vida els carrers” (Improving Life on the Streets), to create superblocks across the city.

The superblock is a new model of mobility that restructures the typical urban road network. With their implementation, superblocks provide solutions to the main problems of urban mobility and improve both the availability and quality of the public space for pedestrian traffic [9]. Superblocks are made up of a grid of basic roads forming a polygon, some 400 m by 400 m, with both interior and exterior components. The interior is closed to motorized vehicles and above ground parking and gives preference to pedestrian traffic. Though the inner streets are generally reserved for pedestrians, they can be used by residential traffic, services, emergency vehicles, and loading/unloading vehicles under special circumstances. The perimeter, or exterior, of superblocks is where motorized traffic circulates, and makes up the basic roads. Superblocks are emerging as an integral solution to the use of public space, uniting urban planning with mobility, and limiting the presence of private vehicles in order to return the public space to the citizen. The importance of the pedestrian is central in the structure of the superblock, such that each grid section has universal accessibility, there is increased safety due to a 10 km/h speed limit, and the habitability and comfort of citizens in public spaces are enhanced [9]. As a result, the implementation of superblocks significantly improves urban quality while reducing the environmental impacts of vehicles. It also increases the quality of life of residents and visitors, enhances social cohesion, and increases economic activity [9].

The “Omplim de vida els carrers” measure aims to improve the habitability of public spaces, progress towards more sustainable mobility, increase and improve urban green, and promote residents’ participation and co-responsibility [8]. Initially, superblocks consisted of amalgams of 3 × 3 blocks, where motorized vehicles and above-ground parking were reduced and preference was given to pedestrian traffic in public space [9]. Currently, due to the difficulty of implementing this plan, superblocks are defined more as larger areas, where the function of each street is recognized and strengthened (approximately 1 in 3 streets is pedestrianized with an expected 20% traffic reduction within the superblock [10]). The superblock process involves creating an action plan through a participatory process, beginning with a small promoting group (representatives of the neighborhood and/or associations with special involvement in the project who act as a link between the technical group and local residents) and ending with the whole neighborhood.

### 1.2. Superblocks Currently Ongoing in Barcelona

**Figure 1:**
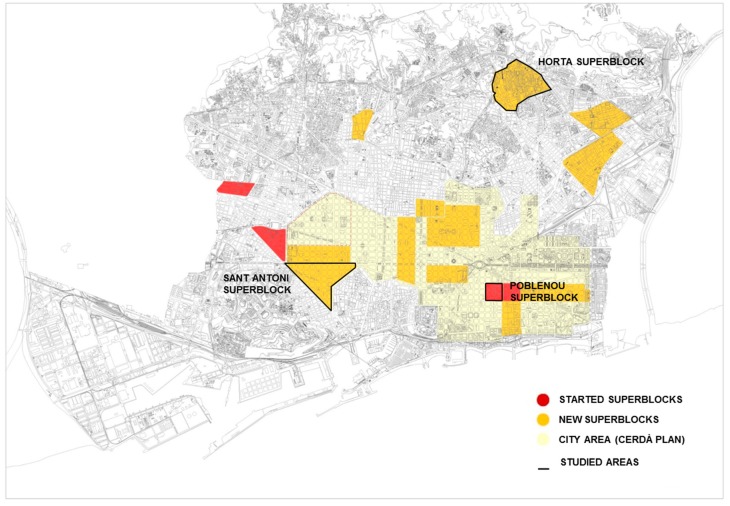
Barcelona superblocks, horizon 2017–2019. Source: Adapted from City-level implementation plan, horizon 2017–2019 in [11].

Currently, five superblocks have been implemented at different stages (Hostafrancs, La Maternitat i Sant Ramón, Poblenou, Sant Antoni, and Horta), and three more are in the participatory process (Girona i entorns, Consell de Cent-Germanetes, and Sant Gervasi de Cassoles). Updated information can be found here (http://ajuntament.barcelona.cat/superilles/ca/). For practical reasons (related to feasibility and resources) this article will focus on three superblocks (marked in black in Figure 1): Poblenou (implemented in 2016), Sant Antoni (first phase implemented in May 2018), and Horta (started work in October 2018).

#### 1.2.1. Poblenou Superblock 

The Poblenou superblock is the typical amalgam of 3 × 3 blocks explained above (see Supplementary Figure S1). However, as it was one of the first superblocks, it was not implemented as ambitiously as planned and, although the inner streets are traffic-calmed with preference given to pedestrians, some parking is still allowed. The superblock comprises the creation of public spaces in some of the former roads and their crossings, including recreational areas (with picnic tables, literary tours, and space for eventual markets), play areas (with several areas devoted to children’s activities), and sports areas (with ping-pong tables, baskets, and an athletics track). 

#### 1.2.2. Sant Antoni Superblock

The first phase of the implementation of the superblock program in the Sant Antoni neighborhood concluded in May 2018 with the urbanization of the Sant Antoni market surroundings. This phase included intervention on two streets (in total four sections of streets forming a cross) and the creation of a new public square in their crossing of 1800 m^2^. In total, 5000 m^2^ of public space was reserved for pedestrians with staying areas for new uses and a greater presence of green space, including trees and bushes (see Supplementary Figure S2).

#### 1.2.3. Horta Superblock

Work on the superblock program in Horta started in October 2018 and is mapped in Supplementary Figure S3. The work mainly included: (1) traffic calming of the main entry street to the neighborhood, with the introduction of leveled surface (no slope in the pavement) and a speed limit of 10 km/h to discourage the entrance of vehicles to the neighborhood; (2) in two streets where there are many private and public equipment and there is almost no sidewalk, the creation of a level surface and removal of parking spaces; and (3) tactical intervention (low budget, temporary, and reversible) with removal of parking spaces and the creation of staying areas in a further street. The works are planned to be finished by December 2019.

### 1.3. The Effect of Urban-Renewal Programs on Health

**Figure 2:**
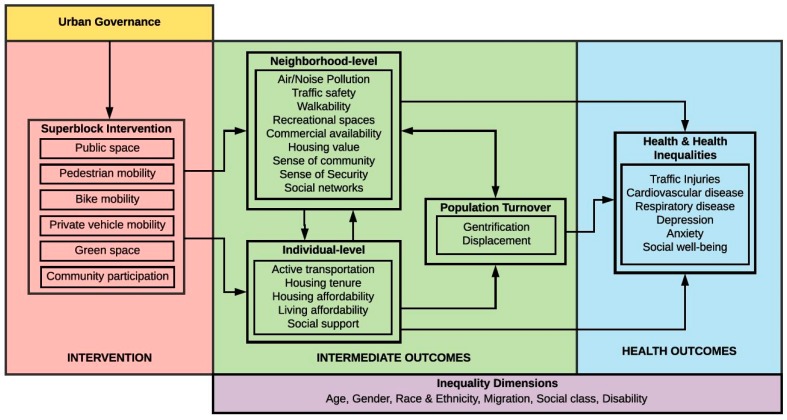
Conceptual framework of the effect of superblocks on health. Source: Mehdipanah R, The effects of superblocks on health and health inequities: a proposed framework for evaluation [12].

In a previous stage of this project, we created a conceptual framework of the possible effects of superblocks on residents’ health based on a literature review (Figure 2) [12]. Briefly, the superblock intervention aims to promote changes in: the public space (improving its use by pedestrians); the different types of mobility (increasing pedestrian and bike mobility and reducing private vehicle mobility); the presence of green areas (increasing and improving urban green and biodiversity); and community participation (working together with residents to design, execute and evaluate the superblocks). These changes are likely to have effects at the neighborhood level, such as a reduction in air and noise pollution and an increase in traffic safety, cycling, and walkability. The increase in the presence of recreational spaces, together with residents’ participation in the whole process, will likely create a higher sense of community and improve social networks while the presence of more people and less cars in the streets will improve the perception of safety. Pedestrian streets also favor commerce and relationships between people in the public space. In addition, some individual-level effects are improvements in active transport and physical activity and social support. However, such improvements can make the neighborhood more attractive, where housing and living affordability can be negatively affected and produce additional effects such as gentrification and displacement. Most of the aforementioned effects can cause effects in health and health inequalities, including improvements in traffic injuries, a reduction in cardiovascular and respiratory diseases, depression, and anxiety, and an enhancement in social well-being. Following this conceptual framework, we discuss the objectives of the project.

### 1.4. Objectives of the Project

The objective of the *Salut als Carrers* (Health in the Streets) project is to evaluate the environmental and health effects of the superblock model with an equity perspective in Barcelona. Specifically:

1. To assess changes in self-reported and mental health and health determinants (physical activity, social support, living affordability, and others) before and after the superblock interventions.
2. To assess whether these changes differ according to socioeconomic characteristics.
3. To assess changes in the levels of air pollutants before and after superblock interventions.
4. To assess the impact of superblocks on road safety.
5. To assess changes in the characteristics, perceptions, and use of public spaces following superblock interventions.
6. To improve health impact assessment (HIA) models on the built environment and superblock interventions.

## 2. Materials and Methods 

This evaluation will employ an integral integrated approach using both quantitative and qualitative methods. The data collection instruments include a health survey, environmental measures, a walkability index, an observation tool to assess physical activity, measures of traffic injuries, and two qualitative studies, one with focus groups and another consisting of guerrilla ethnography. More details for each method are provided below.

Below we describe the studies that will be performed in this project. We have organized this section by superblock, as only some aspects of the studies proposed can be implemented in certain superblocks. More details on the areas covered in each study can be found in Table 1 and Table 2.

### 2.1. Horta Superblock

#### 2.1.1. Health Survey

This sub-study will assess the effect of the superblocks program on people’s health and health behaviors. The sub-study focuses mainly to Objectives 1 and 2. A face-to-face health survey will be conducted in the Horta neighborhood prior to the intervention (May–September 2018) and six months after the end of the intervention (with the same people who completed the survey prior to the intervention). See supplementary material for further details (questionnaire in Spanish). An additional 8 to 10 questions regarding the opinion of the interventions performed in the superblock and the frequency and type of use will be introduced. The sample size will be 1200 people (enough to detect a change of 3% in poor self-reported health [13] and poor mental health (Goldberg scale [14]) with a statistical power of 80% and an α-error of 5%. The sample will be extracted randomly from the municipal register of inhabitants with six age and gender quotas (200 in each quota). Respondents must be individuals living in the neighborhood for at least six months. The survey consists of 141 questions on self-perceived health and quality of life, social support, mental health, mobility, physical activity, neighborhood characteristics, and housing (to detect side effects, such as those of gentrification). McNemar tests will be performed to assess if there are significant changes between the different health outcomes before and after the intervention. If changes are confirmed, further analyses will evaluate whether the effects differ by socioeconomic characteristics such as age, sex, social class, or immigrant status. Poisson regression models with robust variance [15] will be used, where the dependent variable will be an improvement versus no improvement in the health outcome and the independent variables will be the different socioeconomic characteristics.

#### 2.1.2. Environmental Measures

This sub-study will assess the effect of the superblock program in reducing the levels of air pollutants after the intervention. The sub-study relates to Objective 3. Two types of tools will be used for this purpose:

##### (a) Fixed and Mobile Units

Evaluation and characterization of air quality levels in the superblocks are based on measurements performed with the reference methods of the European Union for nitrogen dioxide (NO_2_), particulate matter less than 10 micrometers of diameter (PM_10_), and particulate matter less than 2.5 micrometers of diameter (PM_2.5_). These measurements are performed with a mobile atmospheric control unit owned by the Agència de Salut Pública de Barcelona (Public Health Agency of Barcelona) and with passive diffusers for complementary measures of NO_2_. Results and air pollution profiles (by day and hour) are compared with the other fixed stations of the city surveillance network to control for factors such as meteorological conditions.

The evaluation design will allow the collection of air pollution data before and after the intervention in two different areas of the superblock: (1) in the inner part of the superblock, where measures of traffic calming are likely to reduce pollutant levels; and (2) in the periphery, where pollutant levels could potentially increase given re-routing of the traffic from the inner part of the superblock to the outer part.

The pre- and post-intervention results will be represented in the city general air quality map, which will identify the levels of pollutants (NO_2_ and particles) for each street section of the superblock. In addition, hourly differences in NO_2_ and PM_10_ between the mobile unit and a monitoring station at a fixed location outside the superblock will be calculated. To assess the change in air pollution concentrations after the intervention, the mean of the differences in the post-intervention period will be compared with the mean of the differences in the pre-intervention period by means of *t*-tests or the Mann–Whitney U test depending on the normality of the data.

##### (b) Black Carbon Measures

Black carbon is the sooty black material emitted from motorized transport, coal-fired power plants, and other sources that burn fossil fuels. This material comprises a significant portion of PM. The aim of this part of the project is to measure the black carbon concentrations before and after the superblock intervention in Horta, in three types of pre-defined streets: streets intervened (those where the superblock intervention will be implemented); streets indirectly impacted (those not directly intervened but are closely related to those under intervention and could undergo a change in their traffic or air pollution levels); and unaffected streets (where the traffic and air pollution is unlikely to be affected by the implementation). Two measurement campaigns will be implemented: one before the intervention (May 2018) and another after the intervention. Each measurement campaign will include 10 weekdays of measurements in 21 measurement street points (seven in the streets intervened, called affected; seven in the indirectly impacted, called control-affected; and seven in those not affected, called control; see Figure S3). At each point and day, a 30 minute measurement will be performed on non-rush hours and at the same time of day. The measurements will include measurements of black carbon (using a micro-aethalometer), temperature, humidity, a traffic count, and a street description. A comparative extra measurement point will be implemented in a nearby background fixed station to control for temporal variability. 

#### 2.1.3. Walkability Index 

This sub-study will assess whether the characteristics of the newly organized streets in the superblocks program implemented in Horta will encourage walking by improving walkability indexes. The sub-study relates mainly to Objective 5. Details of streetscapes considered relevant for physical activity will be assessed before (June 2018) and after the superblock interventions with an audit tool named the Microscale Audit of Pedestrian Streetscapes (MAPS) [16]. MAPS is an observational tool that audits the degree of walkability in neighborhoods. It measures “micro” factors of the built environment that influence physical activity levels. These factors are connectivity, residential density, details of the streets (such as pavements and crossings), as well as physical and social characteristics of their design (such as the presence of trees and graffiti). Three different routes, where the superblocks will be implemented and highly used in the neighborhood, will be audited. Two different raters will audit each route to search for assessment validity. Inter-reliability between auditors will be assessed. 

### 2.2. Sant Antoni Superblock

#### 2.2.1. Environmental Measures

As in Horta superblock, NO_2_, PM_10_, and PM_2,5_ environmental measures will be taken with fixed and mobile units.

#### 2.2.2. System for Observing Play and Active Recreation in Communities (SOPARC) Observation Tool

As part of Objective 5 of the study, this sub-study will assess whether the newly developed areas, forming part of the superblock program in the neighborhood of Sant Antoni, encourage physical activity behavior. Specifically, it will do so in: (1) the new Plaça del Mercat, created from a previous crossing of two streets with dense traffic (see new plaza and crossing similar to what it was before in Figure S2); and (2) two sections of the surrounding traffic-calmed streets (the other two only provide access to a car park). This will be done with a systematic observation method named System for Observing Play and Active Recreation in Communities (SOPARC). SOPARC is a validated direct observation tool for assessing physical activity and associated contextual data characteristics in community settings [17]. Two observers (one observing the square and one of its surrounding streets and the other observing the other surrounding street) will carry out weekly observations, four days a week (two weekdays and two weekend days) in four periods of one hour each every day (in our case on weekdays: 8:30–9:30, 12:00–13:00, 15:00–18:00, and 19:00–20:00; and on weekends: 10:00–11:00, 13:30–14:30, 17:00–18:00, and 19:00–20:00). Six weekly observations will be carried out in different seasons across a full year, the first being carried out immediately after the implementation of the superblock. In the last observation, a control site of a crossroad with a nearby market will also be observed. Different patterns of physical activity levels (sedentary, walking, and vigorous) will be described according to the various characteristics of the population (mainly age and sex) and will be compared over time and with the control site.

#### 2.2.3. Guerrilla Ethnography

As part of Objective 5, this qualitative sub-study will complement the quantitative studies carried out in Sant Antoni superblock. Specifically, they will assess citizens’ views and user experience of the new urban space. In particular, we will analyze: (1) overall opinion of the superblocks; (2) strong points of the superblocks, i.e., positive contributions to daily life and quality of life in the neighborhood; (3) weak points or drawbacks; (4) changes in patterns of use of the superblocks; (5) perceived effects on health; and (6) suggested improvements. Differences will be observed when possible by age, type of family (with or without children), functional diversity, racialization, and gender. The spontaneous ethnographic approach or guerrilla ethnography is a type of research that combines ethnographic observation with semi-structured interviews. The guerrillas are carried out by pairs of researchers in sessions of approximately five hours each that take place in the same context of analysis in order to analyze the dynamics occurring in that context. Participants are not previously recruited but are approached spontaneously and are duly informed of the objective and characteristics of the study. Data collection is conducted with multimedia (recording with audio, video, and photographs) that are material for analysis and part of the report. The observation is combined with short-term individual or group interviews. The physical context is especially important, which is why it becomes another item under investigation, and static observation is combined with travelling observation. In the case of the Sant Antoni superblock, three sessions or guerrillas will be performed covering different days and times of the day (Wednesday morning, Friday afternoon, and Sunday morning). The guerrillas will be conducted in an itinerant way, covering the streets of the superblock and the surrounding streets and other places of the neighborhood. 

### 2.3. Poblenou Superblock

#### Qualitative Study with Focus Groups

A qualitative study using focus groups will be carried out to analyze the perception of the people living, studying, or working in the Poblenou neighborhood of the effects of the superblock. The study corresponds mainly to Objectives 1 and 5. The group sessions will have from 6–8 participants and will consist of 60–90 min of discussion with a moderator and an observer. 

Various groups will be formed according to participant characteristics:

1. Fathers or mothers of children living in or near the superblock.
2. Adolescents studying in the superblock.
3. Old people living in or near the superblock.
4. People not living in or near the superblock but studying or working there.
5. Women living in or near the superblock.
6. Other adults not included in the other groups who live in or near the superblock.

Women tend to spend more time in the neighborhood due to their caregiving role and benefit more from urban-renewal programs [18]. For this reason, the script has been reviewed to ensure that it has a gender perspective and has incorporated a group of women only. Otherwise, the groups will be heterogeneous in terms of sex and country of birth. The different issues that the moderator (through dynamization and projection techniques for the promotion of interaction) will raise will be:

- Effects on the use of public spaces (e.g., walking, taking exercise, taking children to play, meeting friends).
- Effects on mobility (e.g., self-mobility, number of cars circulating, noise, air pollution).
- Effects on well-being (e.g., physical health, mental health).
- Effects on social cohesion (e.g., people knew, sense of belonging, participation in associations).
- Effects on the economy (e.g., proximity trade, cost of living).

Sessions will be audio-recorded and transcribed. Thematic analyses will be performed with the support of Atlas Ti software.

### 2.4. All Superblocks

A quasi-experimental evaluation study with a comparison group will be carried out to study the effectiveness of the superblocks in reducing traffic injuries. The intervention group will be the currently implemented superblocks, and the comparison groups will be other areas planned for future superblocks. Data on traffic injuries will be provided by the police, allowing identification of crashes occurring in the intervention and comparison areas as well as their specific location since 2002. A pre–post analysis will be done. Outcome variables will be the total number of traffic injuries, the number of people injured, and the number of pedestrians injured. In both the intervention and comparison areas, relative risks (RR), and their 95% confidence intervals in the post-intervention period versus the pre-intervention period will be estimated using a Poisson-lognormal regression model. The models will be adjusted for traffic volume and street characteristics.

### 2.5. Health Impact Assessment

A recent health impact assessment study has estimated the health implications of a policy scenario related to the full implementation of the superblock model in the city of Barcelona (implementation of 52 superblocks) [19]. This HIA was based on model estimations of how the Superblock model would improve air pollution, noise, green spaces distribution and availability, urban heat islands, transport density, and transport-related physical activity at the city level. These prediction models are the primary sources of uncertainty in the HIAs. In the past, the assessment of such uncertainty has been commonly measured running alternative policy scenarios or using a range of input data in those models. The current protocol provides a tool to assess, in specific locations, real changes produced by the implementation of a superblock, in environmental and transport-related exposures, and offers a unique opportunity to assess the reliability of the HIA models in the built environment and transport sector. This study also provides an additional list of exposures and health outcomes to be considered in future HIA on the superblock model (Supplementary Figure S4). Furthermore, this protocol could also be used as an implementation guide for studies aimed at improving current environmental and transport models used to provide exposure assessments to HIAs. In terms of qualitative study, this will help to add a prioritization of the superblock intervention based on the community vision. This prioritization will support the identification of key exposure pathways and outcomes to be included in the HIA.

## 3. Discussion

### 3.1. Discussion on the Results of the Project

The different superblocks implemented and evaluated in this project differ in characteristics related to the interventions carried out and the implementation process. Poblenou was the first implemented superblock and, at least at the beginning, did not have a participatory process. Poblenou is also a neighborhood that is not very dense in terms of vehicles or population, and people who live there are quite young. Sant Antoni on the other hand, has a more aged population and it is in the middle of Eixample, with a high density of circulating motorized vehicles. The Horta superblock does not create many public spaces but aims to discourage the entrance of private vehicles to the neighborhood and improve pedestrian experience. The choice of methods in each superblock was based mainly on feasibility and adequacy, and we do not expect results and effects measured to be completely similar across superblocks. However, we tried to use as many different methods as possible and complement quantitative with qualitative methods to have more information on the effects observed.

For example, as previously mentioned, the Horta superblock did not create many public spaces, therefore, we do not expect to observe major changes on physical activity or social cohesion. However, changes may be more visible on mobility, air and noise pollution, or the general perception of the neighborhood. Physical activity or social cohesion will also be measured in other superblocks so that results can be compared. Horta was the only superblock in which no interventions had been performed when this project started, therefore the pre- and post-surveys could only be performed there. Regarding air pollution, we already have some evidence that air pollutants, especially NO_2_ have diminished in the Sant Antoni superblock. Air pollution will also be measured in Horta and in future superblocks. Finally, regarding the perception of the superblocks, Poblenou was the first superblock to be implemented and therefore it is likely that some implementation issues were improved in successive experiences. However, we think that we will observe perceived effects on well-being, especially for certain groups such as parents with children and people who work near the superblock that use that space in their free time. In other superblocks (such as Sant Antoni), their use is more generalized and given its situation in the middle of “Eixample” (a place with dense traffic), the perception of increased well-being will be even higher. 

### 3.2. Limitations and Future Research

This project has some limitations. As previously mentioned, when this project started there were several superblocks completed or in progress, therefore pre-intervention measures could not be taken in all superblocks. Also, the participatory process and interventions chosen and implemented vary among superblocks. In some cases, the interventions are small and difficult to show strong health impacts. In addition, each neighborhood has its own unique characteristics, thus results may not be completely comparable between them. However, this project has established different types of methodologies in each superblock so that results can be complemented with each other and eventually applied when there is a further development of the interventions.

In the future, apart from finishing all the aforementioned studies that evaluate health effects of superblocks, key indicators will have to be developed in order to monitor changes in health and quality of life in current and future superblocks. 

### 3.3. Dissemination of Results

The results of the project will be written in the form of reports and scientific articles. We will disseminate the results in press releases as well as in scientific sessions and more general meetings across Barcelona (including some with the superblock promoter groups). In addition, we wish to improve participation by sharing and discussing our results with the study participants, including participants in the survey and the discussion groups. Finally, we will disseminate our results through social networks (Twitter, Instagram, and Facebook), which we have been using throughout the study for information and recruitment of participants.

## 4. Conclusions

This is a protocol to evaluate the impact on health and health determinants of an ambitious urban-renewal program aiming to return public spaces to citizens and improve urban mobility and air quality. Results on the effect on health and health determinants are promising. This study will help to assess the effectiveness of the public policy on health and health inequalities. Evaluation is an important part of public policies in terms of making informed decisions about resource allocation and enhancing public accountability.

## Figures and Tables

**Table 1:** Superblock studied, objectives answered, indicators measured, and sources used in each Quantitative study of the Salut Als Carrers (SAC) project.

Superblock	Sub-Study	Domain	Indicators	Measures
Horta	Survey	General health and well-being	Self-reported health status	pre and post
			Quality of life (EuroQol-5D)	pre and post
			Social support (Duke)	pre and post
		Mental health	Mental health status (General Health Questionnaire, GHQ-12)	pre and post
		Sleep quality	Quantity and quality of sleep	pre and post
		Physical activity	Physical activity (International Physical Activity Questionnaire, IPAQ, short and long)	pre and post
		Mobility	Mobility on working and non-working days according to different modes of transport	pre and post
		Social context	Social cohesion	pre and post
			Satisfaction with the neighborhood (walkability, bikeability, green areas, facilities, traffic, noise)	pre and post
	Mobile Unit	Air pollution	Nitrogen dioxide (NO_2_) measures	pre and post
			Particulate matter (PM) measures	pre and post
			Polycyclic aromatic hydrocarbons (PAHS) measures	pre and post
			Benzene measures	pre and post
	Sensors		Black carbon measures	pre and post
	Walkability Audit	Walkability	Walkability index (Microscale Audit of Pedestrian Streetscapes, MAPS, tool)	pre and post
Sant Antoni	Observation Tool	Use of public spaces	Users’ physical activity levels and modes (System for Observing Play and Recreation in Communities, SOPARC, tool)	post
All Superblocks	Traffic Injuries Register	Traffic injuries	Number of traffic injuries, number of people injured, and number of pedestrians injured by severity and type of vehicle	pre and post

**Table 2:** Superblock studied, objectives answered, issues raised, and sources used in each Quantitative study of the Salut Als Carrers (SAC) project.

Superblock	Source	Domain	Issues Raised
Sant Antoni	Guerrilla ethnography (semi-structured interviews and observation)	General assessment of the superblock	General assessment of the superblock
			Positive aspects of the superblock
			Weak points of the superblock
		Use of public spaces	Patterns of use of the superblock
			Characteristics of the superblock users
			Changes in patterns of use of the superblock
		General health and well-being	Perceived effects on health
			Improvements suggested
Poblenou	Focus groups	Use of public spaces	Perception of effects on the use of public spaces
		Mobility	Perception of effects on own daily mobility
			Perception of effects on circulating vehicles
		Air pollution	Perception of effects on pollution
		Noise pollution	Perception of effects on noise
		General health and well-being	Perception of effects on health and well-being
		Mental health	Perception of effects on mental health
		Social context	Perception of effects on social cohesion
			Perception of effects on the economy

## Supplementary Materials

The following are available online at https://www.mdpi.com/1660-4601/17/8/2956/s1, Figure S1: Interventions performed at the superblock of Poblenou in four categories (recreational areas, play areas, sports areas, and urban renewal locations), Figure S2: Plaça del mercat (newly created plaza in Sant Antoni neighborhood, right) and crossing similar to what it was before (Manso with Viladomat streets, left), Figure S3: Black carbon measurements in the Horta superblock, Figure S4: Health impact assessment (HIA) improvements provided by a pre–post assessment and qualitative study presented in this protocol, Supplementary Material: Survey.
